# Clinicopathological findings and risk factors associated with *Cytauxzoon* spp. infection in cats: A case-control study (2008–2021)

**DOI:** 10.3389/fvets.2022.976173

**Published:** 2022-11-10

**Authors:** Erika Carli, Laia Solano-Gallego, Stefano De Arcangeli, Laura Ventura, Elisa Ligorio, Tommaso Furlanello

**Affiliations:** ^1^San Marco Veterinary Clinic and Laboratory, Padua, Italy; ^2^Departament de Medicina i Cirurgia Animal, Facultat de Veterinària, Universitat Autònoma de Barcelona, Barcelona, Spain; ^3^Department of Statistical Sciences, University of Padova, Padua, Italy

**Keywords:** Cytauxzoonosis, feline, clinical status, laboratory findings, Europe

## Abstract

In Europe, *Cytauxzoon* spp. infection was documented in domestic and wild felids. Cats often develop a subclinical infection, while fatal disease is rare. Currently, information on the epidemiology, risk factors and clinicopathological findings of *Cytauxzoon* spp. infection remains limited and obtained by a single subject or small groups of cats. The objective of this case-control study was to evaluate clinicopathological findings and to describe risk factors associated with *Cytauxzoon* spp. infection in domestic cats. Infected cats (*n* = 39) and non-infected (*n* = 190) cats were selected from the database of the referral San Marco Veterinary Laboratory between 2008 and 2021. Demographic information, a preset questionnaire considering lifestyle, environment, and clinical status, and a CBC performed contextually with the PCR analysis were recorded for all cats. Data on the biochemical profile and serum protein electrophoresis were also evaluated when available. Compared to the control group, infection was more likely to occur in stray cats (24/39, 61.5%, *P* < 0.001), living totally/partially outdoors (36/39, 92.3%, *P* < 0.001), in an urban context (37/39, 94.9%, *P* = 0.002), taken or recently adopted from colonies (34/35, 97.1, *P* < 0.001), with irregular or absent parasite preventive treatments (39/39, 100%, *p* = 0.005), without fleas (28/35, 80%, *P* = 0.047) and without clinical signs (22/39, 56.4%, *p* = 0.026) at the time of medical evaluation. Anemia was not associated with infection, but in cats without clinical signs, the percentage of anemic-infected cats (7/22, 31.8%, *P* = 0.009) was higher compared to non-infected cats (5/65, 7.7%). Furthermore, a decrease in total iron serum concentration approximating the lowest reference interval [median values (IQR): 79 μg/dL (52.25) vs. 50.5 μg/dL (34), *P* = 0.007] was likely in infected cats. No other laboratory findings were associated with infection. Interestingly, a partial/total outdoor lifestyle was a risk factor for infection (OR: 8.58, 95% CI: 2.90–37.0, *P* < 0.001). In conclusion, the present study revealed that *Cytauxzoon* spp. infection manifests itself prevalently as a subclinical infection, based on physical examination and laboratory findings, in domestic European cats. However, subclinical infected cats were more likely to be anemic compared to non-infected.

## Introduction

Cytauxzoonosis is a protozoan tick-transmitted disease that affects wild and domestic felids, traditionally caused by *Cytauxzoon felis*. It is particularly reported in central, south-eastern, and south-central USA (1, 2), although it was described in other parts of the world such as Brazil (3), Iran (4), and China (5). Domestic cats generally develop a severe disease that rapidly progresses to death (6) while wild felids are mostly affected by a subclinical infection that rarely has a fatal evolution (7). However, sick cats that survive *C. felis* infection (8, 9) and subclinical infected (10–13) have been increasingly documented.

In Europe, a piroplasm named *Cytauxzoon* spp. has been reported in both domestic felids (14–16) and wild felids (17–20). The protozoan was molecularly similar, but not superimposable, to *C. felis* (14). The first focus of infection was found in cats living in Trieste in northeastern Italy with a prevalence of 23% (14). Later, *Cytauxzoon* spp. infection has been reported in cats from Portugal (21), France (22), Spain (16), Switzerland (23, 24), Germany (25), and Russia (26). Although sometimes lethal (14, 21, 25), subclinical infection appeared to be more likely and frequent in domestic felines (14, 22, 23). Recently, three *Cytauxzoon* spp. genotypes indicated as *Cytauxzoon europeus, Cytauxzoon otrantorum*, and *Cytauxzoon banethi* were described in European wild cat (*Felis silvestris*) based on 18S rRNA, mitochondrial cytochrome b (CytB) and cytochrome c oxidase subunit I (COI) genes (27). In addition, domestic cats infected with *C. europeus* were documented in Switzerland (24). The available information on the epidemiology and clinicopathological findings of *Cytauxzoon* spp. infection in Europe is currently limited and often based on the evaluation of a single subject or of small groups (21–23, 28).

Given this context, the case-control study reported here was aimed at (1) evaluating clinicopathological findings and (2) describing risk factors associated with *Cytauxzoon* spp. infection in domestic cats in Italy.

## Materials and methods

### Study design and setting

This case-control study compared cats with and without *Cytauxzoon* spp. infection. Data were extracted from the database of PCR analysis performed at the San Marco Veterinary Laboratory (SMVL) referred from May 2008 to July 2021. The source database included the records of blood molecular analysis of *Cytauxzoon* spp. obtained from (1) cats published in previous works (14, 15) (*n* = 120) and (2) cats collected by clinicians distributed throughout Italy during routine veterinary procedures or illnesses, with or without suspected *Cytauxzoon* spp. infection (*n* = 526). The SMVL provides services to veterinary clinics spread throughout Italy. Approximately 1,400 molecular analyzes per year are performed in feline samples at SMVL.

A focus of *Cytauxzoon* spp. (14) was previously described in Trieste (45°39′01″ N, 13°46′13″ E), a seaport city in northeastern Italy. It is located in the northern part of the high Adriatic Sea near the border with Slovenia. It is characterized by a long coastline bordered by grasslands, forests, and karstic areas (https://en.wikipedia.org/wiki/Trieste). Ticks (29) and wild felids such as Eurasian Lynx (*Lynx lynx*) and the wild cat (*Felis silvestris*) (20) are reported in the region named Friuli Venezia Giulia, where Trieste is located.

### Patient selection, inclusion criteria, sampling, questionnaire, and variable characteristics

**Figure 1:**
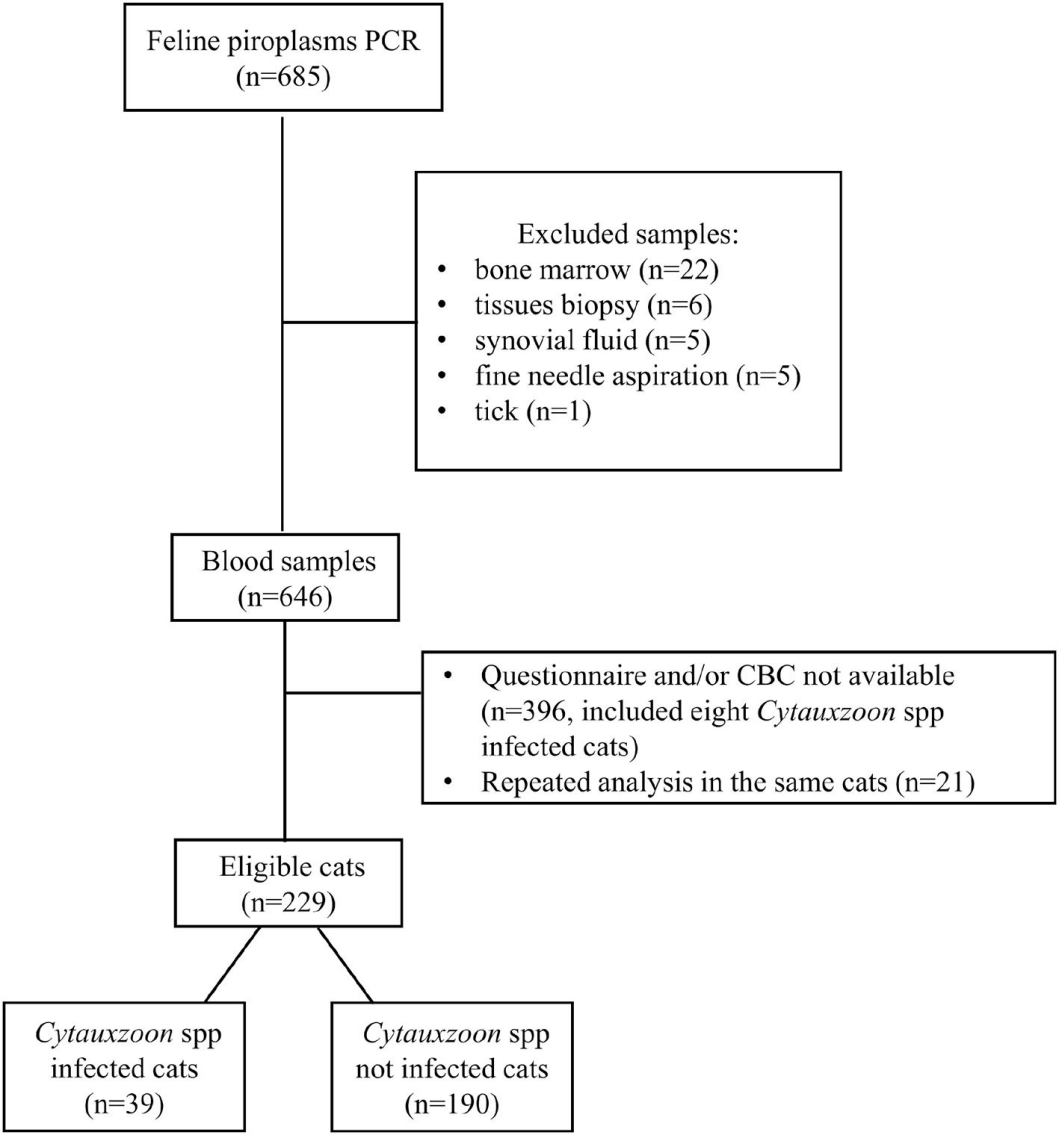
Diagram of the selection of the cats included in the study.

The selection of cats included in the present study is shown in Figure 1. The inclusion criteria were the following: (1) a blood piroplasm PCR analysis performed on the 18S rRNA gene, (2) a preset questionnaire, and (3) a CBC performed contextually to the PCR analysis.

Therefore, according to the inclusion criteria described above, the studied data set included random healthy/unhealthy, stray/owned (1) cats living in the Trieste focus (*n* = 90) (14, 28), and (2) cats (*n* = 139) living in the north (*n* = 112), in the center (*n* = 20), and in the south (*n* = 7) of Italy.

Based on the results of the PCR analysis, eligible cats were assigned to *Cytauxzoon* spp. infected (case) or not infected (control) groups. For the purposes of the case definition, 18S rRNA gene sequencing was performed randomly for the identification of piroplasm species in cats living in Trieste (14, 28), while it was considered mandatory for the others. The unmatched control group involved cats enrolled between 2008 and 2011, while cases were obtained throughout the study period.

Blood samples were taken from stray cats that lived in urban Trieste colonies as part of spay/neuter programs and from owned cats that underwent routine blood screening for various medical reasons.

The clinicians completed a multiple choice questionnaire at the same time the blood sample was collected. The questionnaire concerned patient characteristics (age, sex, breed), type of cat (owned or stray), lifestyle (indoor or partially/total outdoor), environment (rural/city), type of housing (taken/recently adopted from colony or cattery/household), parasite preventive treatments (regular or irregular/absent), ticks and/or flea presence and detection of clinical signs at the time of medical examination. The results of the questionnaire were already available on the cats observed between 2008 and 2011. The questionnaire of infected cats diagnosed subsequently was retrieved through a telephone call from one of the authors (EC). Only questionnaires based on clinical records collected by clinicians at the time of sampling were considered suitable.

When available, the biochemical profile and serum protein electrophoresis performed at the same time as the other assays were also included.

All samples were collected solely for the cat's health benefit.

When the age of the cats was not available, the clinicians provided an estimated age value. For this reason, this variable was not considered reliable for statistical analysis.

Cats were also stratified according to their geographical origin (north, central, or southern Italy) and the focus of Trieste. The dichotomic variable “anemia” was created using the red blood cell (RBC) concentration value of 6.35 × 10^6^/μL (lower limit of the reference interval of SVML) rather than using hematocrit (Hct) as discriminator, to avoid possible incorrect classification due to storage artifacts (30).

### The analytic methods

All clinicopathological tests were performed at the SMVL within 24–36 h after collection. The CBC was performed on K3EDTA whole blood samples using automatic cell counters (ADVIA^®^ 120, ADVIA^®^ 2120, and ADVIA^®^ 2120i Hematology System, Siemens Healthineers GmbH, Erlangen, Germany) and was always accompanied by a microscopic evaluation of the blood smear. The biochemical profile was assessed on serum samples using an automated analyzer (Olympus AU2700, Olympus Diagnostic, Hamburg, Germany and Atellica CH930, Siemens Healthineers, GmbH, Erlangen, Germany). Serum protein electrophoresis was performed using Capillarys 1 and Capillarys 2 (Sebia, Florence, Italy).

Laboratory parameters evaluated included the following: RBC, hemoglobin, Hct, mean cell volume, mean corpuscular hemoglobin concentration, white blood cell concentration, neutrophils, lymphocytes, monocytes, eosinophils, platelet count (PLT), mean platelet volume, creatine kinase, aspartate aminotransferase, alanine aminotransferase, alkaline phosphatase, γ−glutamyltransferase, total bilirubin, total protein, albumin, globulin, cholesterol, triglycerides, blood urea nitrogen, creatinine, glucose, calcium, magnesium, phosphorus, sodium, potassium, total iron (Fe), serum amyloid A, albumin (by serum protein electrophoresis), α-globulin, α_1_-globulin, α_2_-globulin, β-globulin, and γ-globulin.

### Molecular analysis, sequencing, and phylogenetic analysis

The molecular investigation was performed by (1) panpiroplasm end-point PCR, followed by sequencing of amplicons for species identification, carried out throughout the study period and used for the detection of *Cytauxzoon* spp. with a diagnostic purpose, (2) a phylogenetic analysis evaluated on the sequences of the nearly complete 18S rRNA of the stored samples still available at the time of the conceptualization of the study.

DNA was obtained from 200 μL of blood from each cat using the High Pure PCR Template Preparation Kit (Roche Diagnostics GmbH, Mannheim, Germany). Blood was incubated with 40 μL of proteinase K and 200 μL of binding buffer at 72°C for 1 h. Subsequent steps were performed according to the manufacturer's instructions. DNA was eluted in 50 μL of elution buffer. Molecular detection of piroplasms was carried out using a conventional PCR assay using the sense primer PIROA (5′-AATACCCAATCCTGACACAGGG-3′) and the antisense primer PIROB (5′-TTAAATACGAATGCCCCCAAC-3′) that amplify a fragment of ~412 bp of the 18S rRNA gene (31). PCR reactions were carried out in a final volume of 25 μL containing 2.5 μL of DNA template, 0.4 μM of each primer, 1.8 mM of MgCl2, 200 μM of each dNTP, 2.5 U of FastStart High Fidelity Enzyme, and 1X of FastStart High Fidelity Reaction Buffer (Roche Diagnostics GmbH, Mannheim, Germany). The thermal cycling conditions consisted of an initial denaturation at 95°C for 2 min, 35 cycles of denaturation at 95°C for 30 s, annealing at 58°C for 30 s and extension at 72°C for 30 s, followed by a final extension step at 72°C for 5 min. Positive controls and negative controls (water) of the PCR were included in each run. All reactions were performed using an automated thermal cycler (Applied Biosystems 2720, Carlsbad, CA, USA). The amplicons were visualized by UV transillumination after electrophoresis of 5 μL of the reaction solution in a 2.2% agarose FlashGel DNA cassette (Lonza, Rockland, ME, USA). All PCR products containing expected size amplicons were submitted to an external laboratory for direct sequencing on both strands (BMR Genomics srl, Padua, Italy). The nucleotide sequences obtained were compared with those available in the GenBank database using the BLAST program (32) for species identification.

To perform phylogenetic analysis, *Cytauxzoon* spp. positive DNA extracts from −20°C stored still available samples were subjected to amplification and sequencing of the nearly complete 18S rRNA gene as previously described (33). The contigs were assembled and edited using BioEdit 7.2.5 software, and the sequences obtained were aligned with a set of 34 relevant corresponding sequences of *Cytauxzoon* spp. longer than 1,000 bp retrieved from GenBank. Alignment was carried out using the MUSCLE algorithm (34) implemented in MEGA version 11 (35) and a maximum likelihood (ML) tree was inferred with the same software. Gaps or missing data in the alignment were deleted and the substitution model with the lowest Bayesian information criterion (BIC), calculated using the specific function of MEGA 11, was selected. The reliability of the tree was evaluated by performing 1,000 bootstrap replicates.

The nucleotide sequences obtained in this study were submitted to the GenBank database under accession numbers OM004051-OM004057.

### Statistical analysis

Statistical analysis was performed using R for MacOS (version 4.1.0). After rejecting the normality assumption for quantitative variables with the Shapiro-Wilk test, quantitative data were expressed as medians and interquartile ranges (IQR). Categorical variables were expressed as counts and percentages in each category. The differences between the infected *Cytauxzoon* spp. and the control groups were analyzed in bivariate analysis with the Mann-Whitney rank sum for quantitative variables and with Pearson's chi-square test with Yates continuity correction or Fisher's exact test with categorical variables.

Two sub datasets that included subclinical and sick cats, respectively, were created to better assess the role of the anemic status during infection. Categoric variable anemia was analyzed referring to the presence/absence of *Cytauxzoon* spp. in each sub dataset.

The percentages of dichotomous variables such as sex, breed, type of cat, lifestyle, environment, ticks, fleas, clinical signs, and anemia that occur in the infected cat group were analyzed using the one sample proportion test considering a true proportion not equal to 0.5 as an alternative hypothesis. For each estimated proportion, the 95% confidence interval (95% CI) was calculated to provide a range of values that is likely to include the proportion of the real population of each variable.

To identify the risk factors for *Cytauxzoon* spp. infection, a multivariable logistic regression model was estimated, after backward variables selection. The presence of anemia was included in the model due to its clinical relevance during infection. The results of the fitted logistic regression were expressed as odds ratio (OR), 95% CI and *p*-value.

The statistical significance was declared for *P* < 0.05.

## Results

### Case and control cats

During the evaluation period, piroplasm PCR analysis was performed in 646 feline blood samples (Figure 1), with a diagnosis of *Cytauxzoon* spp. infection in 47 cats (7.3%).

Four hundred and seventeen cats were excluded for various reasons. Finally, 229 cats met the inclusion criteria and, according to the PCR results, were divided into case (infected, *n* = 39) and control (non-infected, *n* = 190) groups (Figure 1).

**Figure 2:**
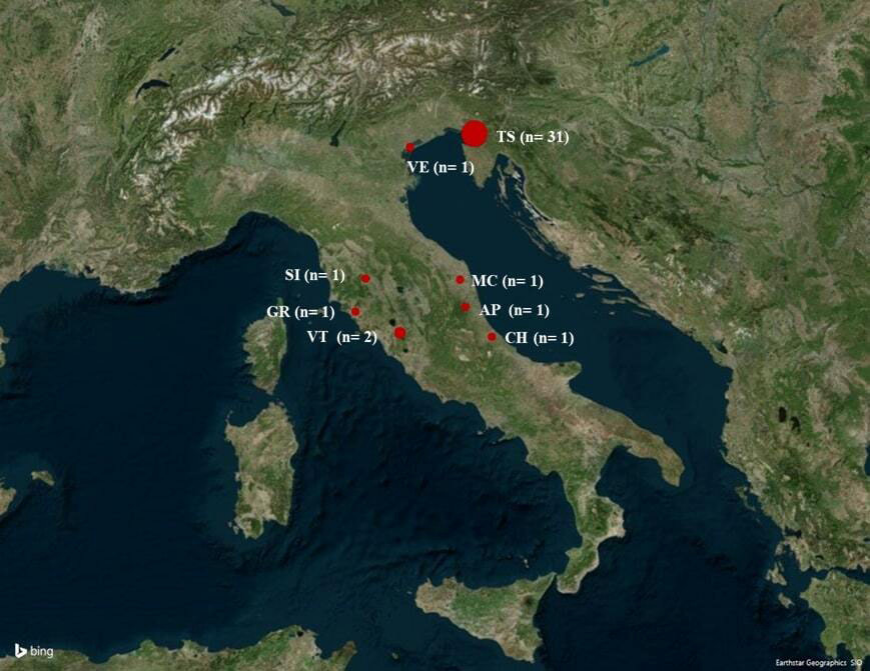
Map of Italy showing the cities where *Cytauxzoon* spp. infected cats were diagnosed. The map was obtained by Microsoft 3D Maps in Excel 2016 software. AP, Ascoli Piceno; CH, Chieti; GR, Grosseto; MC, Macerata; SI, Siena; TS, Trieste; VE, Venezia; VT, Viterbo.

*Cytauxzoon* spp. infected cats were detected in the northeast (*n* = 32, 82.1%) and in the center (*n* = 7, 17.9%) of Italy (Figure 2).

**Figure 3:**
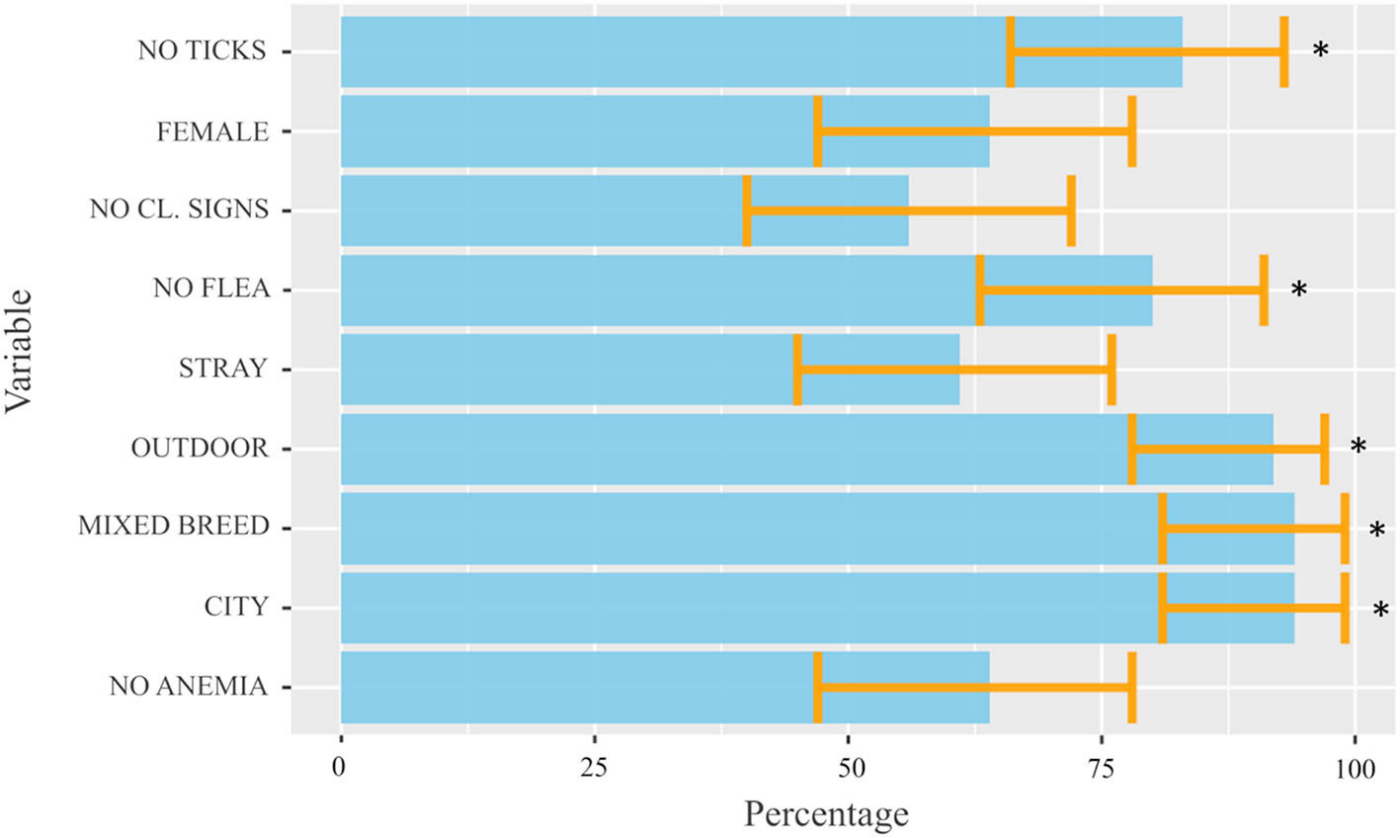
Estimated proportions for each dichotomous variable obtained by the one-sample proportional test from cats infected with *Cytauxzoon* spp. included in the study. The light blue bars represent the estimated proportions for each dichotomous variable obtained by the one-sample proportional test, considering a true proportion not equal to 0.5 as an alternative hypothesis. The orange lines (error bars) represent the width of the 95% IC calculated for each variable and provide interval estimations (and the uncertainty of the measurement) for the real population proportion of each variable. The *P*-value < 0.05 was considered significant and was indicated by *. CL, clinical.

The demographic information and clinical characteristics of the case and control groups are summarized in Table 1. The cats infected with *Cytauxzoon* spp. were predominantly female (25/39, 64.1%) and mixed breed (37/39, 94.9%) with a median age (IQR) of 24 months (60). Compared to the control group, infection was more likely to occur in stray cats, living partially/total outdoors, in an urban context, taken or recently adopted from colonies, with irregular or absent parasite preventive treatments, without fleas and without clinical signs at the time of the medical evaluation. By the one sample proportional test the *Cytauxzoon* spp. infected cat population was likely represented by high percentages of mixed breed cats (94.9%, 95% CI: 81.4–99.1%, *P* < 0.001) that live prevalently or totally outdoors (92.3%, 95% CI: 78–98%, *P* < 0.001) in urban areas (94.9%, 95% CI: 81.4–99.1%, *P* < 0.001), with no presence of flea (80%, 95% CI: 62.5–90.9%, *P* < 0.001) and ticks (82.9%, 95% CI: 65.7–92.8%, *P* < 0.001) at the time of clinical examination (Figure 3).

**Table 1:** Characteristics of patients at baseline in the total study population and in the case and control groups.

**Variable**	**Overall cats**	**Cases**	**Controls**	***P-*value**
**Age, months, (*****n*** **=** **229)**, median (IQR)	60 (132)	24 (60)	73.50 (126)	ND
**Sex (*****n*** **=** **223)**				
Male (%)	87 (39)	14 (35.9)	73 (39.7)	0.796
Female (%)	136 (61)	25 (64.1)	111 (60.3)	
**Breed (*****n*** **=** **229)**				
Mixed breed (%)	205 (89.5)	37 (94.9)	168 (88.4)	0.387
Purebred (%)	24 (10.5)	2 (5.1)	22 (11.6)	
**Type of cat (*****n*** **=** **229)**				
Owned (%)	157 (68.6)	15 (38.5)	142 (74.7)	< 0.001
Stray (%)	72 (31.4)	24 (61.5)	48 (25.3)	
**Lifestyle (*****n*** **=** **226)**				
Indoor (%)	72 (31.9)	3 (7.7)	69 (36.9)	<0.001
Partially/total outdoor (%)	154 (68.1)	36 (92.3)	118 (63.1)	
**Living area (*****n*** **=** **229)**				
North (%)	202 (88.2)	32 (82.1)	170 (89.5)	0.057
Center (%)	20 (8.7)	7 (17.9)	13 (6.8)	
South (%)	7 (3.1)	0	7 (3.7)	
**Living in the TS focus area (*****n*** **=** **229)**				
No (%)	139 (60.7)	8 (20.5)	131 (68.9)	<0.001
Yes (%)	90 (39.3)	31 (79.5)	59 (31.1)	
**Environment (*****n*** **=** **220)**				
Rural (%)	57 (25.9)	2 (5.1)	55 (30.4)	0.002
City (%)	163 (74.1)	37 (94.9)	126 (69.6)	
**Housing type (*****n*** **=** **164)**				
Colony (%)	91 (55.5)	34 (97.1)	57 (44.2)	<0.001
Cattery, family (%)	73 (44.5)	1 (2.9)	72 (55.8)	
**Preventive treatment of parasites (*****n*** **=** **229)**				
Regular (%)	38 (16.6)	0	38 (20)	0.005
Irregular/absent (%)	191 (83.4)	39 (100)	152 (80)	
**Ticks presence (*****n*** **=** **216)**				
Absent (%)	185 (85.6)	29 (82.9)	156 (86.2)	0.802
Present (%)	31 (14.4)	6 (17.1)	25 (13.8)	
**Fleas presence (*****n*** **=** **218)**				
Absent (%)	139 (63.8)	28 (80)	111 (60.7)	0.047
Present (%)	79 (36.2)	7 (20)	72 (39.3)	
**Clinical signs (*****n*** **=** **221)**				
Absent (%)	87 (39.4)	22 (56.4)	65 (35.7)	0.026
Present (%)	134 (60.6)	17 (43.6)	117 (64.3)	
**Anemia (*****n*** **=** **229)**				
Absent (%)	174 (76)	25 (64.1)	149 (78.4)	0.089
Present (%)	55 (24)	14 (35.9)	41 (21.6)	

The infected group with *Cytauxzoon* spp. (case) and not infected (control) were compared using the Pearson's chi-square test with Yate's continuity correction or Fisher's exact test for categorical variables. The *P*-value < 0.05 was considered significant.

IQR, Interquartile Range; TS, Trieste; ND, Not Determined.

Anemia was present in 14/39 (35.9%) of infected cats and was not associated with infection compared to the control group (41/149, 21.6%, *P* = 0.089). When the sub dataset of cats without clinical signs was analyzed at the time of diagnosis (*n* = 87), the percentage of anemia was likely higher in cats infected with *Cytauxzoon* spp. compared to those not infected (7/22, 31.8% infected vs. 5/65, 7.7% not infected, *P* = 0.009). In the subgroup of cats that presented clinical signs at the time of diagnosis (*n* = 134), anemia was not associated with infection (7/17, 41.2% infected vs. 36/117, 30.8% not infected, *P* = 0.401). The clinical signs observed by clinicians for cats infected with *Cytauxzoon* spp. were a non-specific (i.e., neurological signs, slimming, diarrhea, stomatitis, dermatitis, among others).

Laboratory findings are shown in Table 2. Compared to the control group, cats infected with *Cytauxzoon* spp. showed a higher platelet concentration [median values (IQR): 357 × 10^3^/μL (227) vs. 288 × 10^3^/μL (172.5), *P* = 0.013] and a lower total iron [median values (IQR): 50.5 μg/dL (34) vs. 79 μg/dL (52.25), *P* = 0.007]. No other statistical differences were observed with respect to laboratory findings.

**Table 2:** Laboratory findings of all cats and infected and non-infected *Cytauxzoon* spp. groups.

**Laboratory parameters (reference**	**Overall cats**	**Cases**	**Controls**	***P-*value**
**interval, unit)**	**median (IQR)**	**median (IQR)**	**median (IQR)**	
**CBC**	(*n* = 229)	(*n* = 39)	(*n* = 190)	
RBC (6.35–9.5 × 10^6^/μL)	7.54 (2.19)	6.90 (3.05)	7.65 (2.01)	0.263
HB (9.6–14.3 g/dL)	10.90 (3.1)	10.80 (3.75)	10.90 (2.95)	0.298
HCT (28.0–42.5%)	34.60 (10.4)	33.90 (13.4)	34.60 (10.38)	0.150
MCV (38.0–49.5 fL)	45.20 (6.78)	45.30 (8.05)	45.15 (6.73)	0.941
MCHC (31.0–35.0 g/dL)	31.20 (2.6)	31.70 (5.4)	31.00 (2.48)	0.260
WBC (5.0–11.0 × 10^3^/μL)	11.47 (7.22)	12.82 (5.74)	10.96 (7.15)	0.097
Neutr (2,500–7,000/μL)	7,027 (6,247)	8,182 (5,557)	6,950 (6,299.25)	0.151
Lymph (1,300–5,500/μL)	2,520 (2,014)	2,556 (2,593)	2,407 (1,863.75)	0.390
Mon (65–250/μL)	306 (363)	389 (483.50)	293 (359)	0.085
Eos (70–800/μL)	540 (520)	610 (504)	521.50 (539.25)	0.094
PLT (130–430 × 10^3^/μL)	296 (193)	357 (227)	288 (172.5)	0.013
MPV (7.9–17.5 fL)	16.60 (5.20)	18.10 (4.80)	16.30 (4.95)	0.054
**Biochemical profile**	*n* = 157	*n* = 13	*n* = 144	
CPK (90–320 IU/L)	140.50 (136.75)	198 (148.25)	140 (129.25)	0.181
AST (15–35 IU/L)	31.50 (23.50)	24.50 (22.25)	32 (23.50)	0.502
ALT (32–87 IU/L)	57 (52.5)	48 (28.5)	58 (51.75)	0.220
ALP (19–70 IU/L)	24 (16–39)	22.50 (21.75)	24 (15–39)	0.926
GGT (0.1–0.6 IU/L)	0.50 (0.72)	0.50 (1)	0.50 (0.72)	0.563
TB (0.14–0.26 mg/dL)	0.21 (0.10)	0.21 (0.10)	0.21 (0.10)	0.272
TP (6.3–7.8 g/dL)	7.40 (1.20)	7.20 (0.70)	7.45 (1.30)	0.473
Albumin (3.0–4.0 g/dL)	3.0 (0.7)	3.0 (0.7)	3.0 (0.63)	0.846
Globulin (3.0–4.5 g/dL)	4.4 (1.2)	4.1 (1)	4.4 (1.2)	0.536
Cholesterol (95–210 mg/dL)	145 (65.25)	148.50 (93)	145 (64.75)	0.583
Triglycerides (19–81 mg/dL)	51 (46.5)	54 (59.5)	51 (45.25)	0.561
BUN (32–64 mg/dL)	59 (30)	53 (46)	59 (29.25)	0.367
Creatinine (0.95–1.85 mg/dL)	1.51 (0.71)	1.40 (0.6)	1.52 (0.72)	0.295
Glucose (86–116 mg/dL)	118 (56.5)	130.5 (44.25)	117 (58.5)	0.487
Ca (9.3–11.2 mg/dL)	9.5 (1)	9.6 (1.57)	9.5 (1)	0.590
P (3.5–6.6 mg/dL)	4.5 (1.8)	5.0 (2.47)	4.5 (1.82)	0.216
Na (145–152 mEq/L)	151 (5)	150 (3.48)	151.5 (4.25)	0.056
K (3.5–4.7 mEq/L)	4.10 (0.8)	4.45 (0.50)	4.00 (0.73)	0.013
Mg (0.81–1.05 mmol/L)	0.90 (0.15)	0.90 (0.18)	0.90 (0.15)	0.976
Total iron (50–118 μg/dL)	77.50 (57)	50.50 (34)	79 (52.25)	0.007
SAA (0.1–0.5 μg/mL)	0.40 (29.30)	0.70 (1.45)	0.40 (33.10)	0.812
**Serum protein electrophoresis**	*n* = 154	*n* = 12	*n* = 142	
Albumin (39.4–54.8%)	49.80 (12.04)	48.35 (6.67)	49.85 (12.65)	0.340
Alpha globulin (17.8–27.6%)	16.85 (6.34)	16.75 (4.28)	17 (6.6)	0.893
Alpha_1 globulin (0.8–1.6%)	1.80 (1)	1.85 (1.30)	1.80 (1)	0.721
Alpha_2 globulin (17.0–26.0%)	14.85 (5.62)	14.50 (3.43)	14.90 (6)	0.726
Beta globulin (6.4–9.4%)	11.40 (3.54)	11.30 (2.38)	11.40 (3.64)	0.981
Gamma globulin (16.2–28.2%)	20.15 (10.88)	23.20 (9.18)	20 (10.95)	0.467

Quantitative variables, not normally distributed by the Shapiro-Wilk test, were reported as median and interquartile range (IQR). *Cytauxzoon* spp. infected and non-infected groups were compared by the Mann-Whitney rank sum test. The *P*-value < 0.05 was considered significant.

IQR, interquartile range; CBC, Cell Blood Count; RBC, Red Blood Cell count; HB, hemoglobin; HCT, hematocrit; MCV, mean cell volume; MCHC, mean corpuscular hemoglobin concentration; WBC, white blood cell count; Neutr, neutrophils; Lymph, lymphocytes; Mon, monocytes; Eos, eosinophils; PLT, platelet count; MPV, mean platelet volume; CK, creatine kinase; AST, aspartate aminotransferase; ALT, alanine aminotransferase; ALP, alkaline phosphatase; GGT, γ−glutamyltransferase; TB, total bilirubin; TP, total protein; BUN, blood urea nitrogen; Ca, calcium; Mg, magnesium; P, phosphorus; Na, sodium; K, potassium; SAA, serum amyloid A.

In the multivariable analysis *Cytauxzoon* spp. infection was strongly associated with a partially/total outdoor lifestyle (OR: 8.58, 95% CI: 2.9–37, *P* < 0.001) and living in urban areas (OR: 11.9, 95% CI: 3.38–76.7, *P* = 0.001). The categorical variable anemia included in the logistic model has shown the following OR: 2.22, 95% IC: 0.97–5.05, *P* = 0.056. The AUC (area under the curve) of the model was 0.78 (95% IC: 0.71–0.85).

### Sequencing and phylogenetic analysis

The DNA amplicon sequences obtained for diagnostic purposes based on pan-piroplasm end point PCR (31, 33) of cats living in the Trieste focus (*n* = 13) and in other parts of Italy (*n* = 8) showed a nucleotide identity >99% with reference sequences from *Cytauxzoon* spp. available in GenBank.

Sequencing of the nearly complete 18S rRNA gene was successful in seven cases.

**Figure 4:**
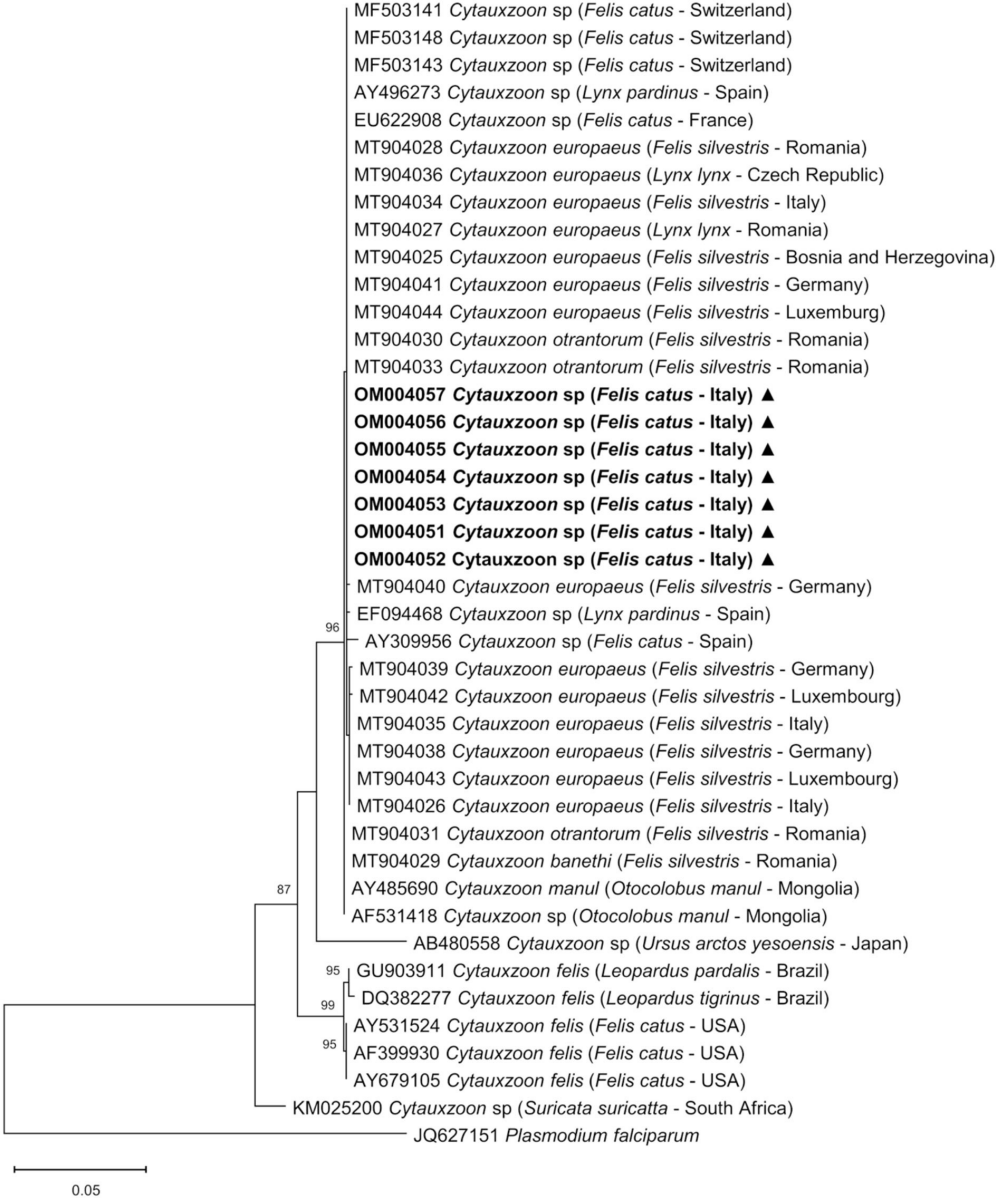
Phylogenetic relationship of *Cytauxzoon* spp. inferred from an alignment of 979 sites of the 18S rRNA gene. The maximum likelihood nucleotide tree inferred from an alignment of 979 sites of *Cytauxzoon* spp. 18S rRNA gene sequences using the Tamura 3-parameter substitution model with discrete gamma distribution. The GenBank accession number, species, host, and country of origin are included for each *Cytauxzoon* spp. sequence. The nucleotide sequences generated in this study are in bold and marked with a triangle. *Plasmodium falciparum* was included in the analysis as an out group. Bootstrap values <70% are indicated in the respective branches.

The ML phylogenetic tree (Figure 4) was estimated at 979 sites, due to the complete deletion of gaps and missing data in the alignment; the Tamura 3-parameter (35) nucleotide substitution model with discrete gamma distribution showed the lowest BIC and was selected.

Phylogenetic analysis showed that the seven 18S rRNA piroplasmid gene sequences obtained in this study grouped with other European *Cytauxzoon* spp. from domestic and wild felids and with two sequences of *C. manul* detected in Pallas' cats from Mongolia, while *C. felis* sequences from North and South America grouped together in a clearly distinct cluster.

## Discussion

To the best of the authors' knowledge, this is the first case-control study and, until now, the most substantial work on demographic and clinical data on *Cytauxzoon* spp. infection in domestic cats carried out in Europe. In fact, apart from the first description of the Trieste focus (14) and a recent paper mainly focused on the epidemiology and genetics of the parasite (24), most of the papers available in domestic cats are single case reports (21, 22, 25) or descriptions of a small group of infected subjects (16, 23, 28, 36).

Although in the present study a wide range of hematological and biochemical parameters were analyzed, few laboratory findings were statistically associated with *Cytauxozoon* spp. infection, with values not exceeding the reference interval. Infection appears more likely in cats without clinical signs and with alterations in few or absent laboratory parameters at the time of diagnosis. Therefore, this study supports, from a laboratory perspective, the idea that *Cytauxzoon* spp. infection in Italy is often subclinical (14, 28), as is occasionally the case for infection with *C. felis* in the USA (10, 11, 37, 38).

At a significance level of 0.05, anemia does not appear to be associated with *Cytauxzoon* spp. However, it should be noted that the *P*-values obtained for this categorical variable are borderline for significance by both the Pearson chi-square test and the multivariable logistic regression. Furthermore, while for the most part non-anemic, subclinical infected cats showed a significantly higher percentage of anemia compared to those not infected. It could be reasonable that in apparently healthy infected cats, parasitized erythrocytes are removed from the bloodstream or hemolyzed, giving rise to a variable rate of reduction of RBC that is not necessarily so marked that it evolves in an anemic state. Furthermore, considering the low concentration of iron associated with this infection, the mechanism of anemia of inflammatory disease (39) could be another explanation for our findings. It is important to note that anemia needs to be further investigated, by adding more subjects to the sample and evaluating the presence/absence of regeneration, to confirm and understand its real role and the mechanisms involved during *Cytauxzoon* spp. infection in both subclinical and sick cats.

Although many biochemical parameters were evaluated in the present study, even if just above the minimum value, only the lower iron concentration observed in the case group seems to be clinically relevant compared to the control group. Hypoferremia could be caused by a shift of iron to the storage site (e.g., inflammation disease) while other factors, such as the young age of the animals or Fe deficiency (39) appeared to be less likely. It is important to note that the biochemical and serum electrophoresis parameters were obtained from a lower number of cats compared to the CBC parameters. Data from more subjects might be needed to find mild differences between infected and uninfected cats.

In the present study, living partially or completely outdoors was strongly associated with *Cytauxzoon* spp. infection. Additionally, infection was more likely in stray cats. These results agree with previous works on both *Cytauxzoon* spp. (14, 16, 28) and *C. felis* (4, 40). They suggest that this lifestyle is a risk factor for *Cytauxzoon* spp. infection. Outdoor cats could be exposed to the sources of infection traditionally reported for *C. felis* as the tick vectors (6, 41) and wild felids as reservoirs (42). However, to our knowledge, the highly competent vectors *Amblyomma Americanum* (41, 43) and *Dermacentor variabilis* (44) that experimentally transmitted *C. felis* are not found in Europe. Furthermore, *Cytauxzoon* spp. has not yet been isolated from ticks collected in European countries (36, 45). However, *Cytauxzoon* spp. was isolated from European wild cats (*Felis silvestris*) in Italy (20), Romania (19), Bosnia and Herzegovina (46), and France (24) as well as in the Eurasian lynx (*Lynx lynx*) in Romania (19) and in the Iberian lynx (*Lynx pardinus*) in Spain (17, 18). Domestic cats and wild cats or European lynx are considered to live in sympatry, inbreed, and share pathogens (20, 47). Consistent with previous findings, the infected cats reported in the present work lived interestingly in the north-east and central Italy in areas almost overlapping those in which *Cytauxzoon* spp. was isolated from wild cats (20). These findings reinforce the possible role of the wild felid as a reservoir for this infection in Italy.

However, the frequent subclinical *Cytauxzoon* spp. infection described in the present work is consistent with previous studies (6, 9, 12, 14, 40) and contributes to assigning to apparently healthy infected cats the role of “long-life domestic reservoir” for this parasite. This role appears to be more important than the one played by wild felids, due to the greater possibility of contact between infected and non-infected domestic cats. Thus, *Cytauxzoon* spp. could be introduced more easily in a geographic area where it was not endemic, for example, during owner relocation (12) or after the adoption of an infected subject from a colony or a cattery. More studies are needed to determine the dynamics of the spillover of *Cytauxzoon* spp. from felines in sylvatic ecosystems to domestic cats in urban locations, the role of domestic infected cats as reservoirs, and if there is another potential competent vector for this infection.

Cats living in an urban area appeared to be strongly associated with *Cytauxzoon* spp. compared to other settings (rural, hilly, home). This result disagrees with previous papers reporting that *C. felis* and *Cytauxzoon* spp. are more likely in outdoor cats, especially in low-density residential or rural areas where contact with ticks and wild felids might be more frequent (16, 48). It is interesting to note that, in the present study, cats living in the colony, at the time of evaluation or recently adopted from it, were more likely to be infected by *Cytauxzoon* spp. than cats living in the cattery or family. As most of the colony cats were from the Trieste urban area focus (14, 28), a bias referring to the result of the urban environment could be suspected. Consequently, it is likely that our observations might mainly reflect the characteristics of a particular habitat as a colony rather than the context of the urban environment. In colonies, the interaction of cats with each other or with other animals (as wild felids) or a vector appears possibly more like a “natural” condition. This could lead to some reflections on the pathogenesis of *Cytauxzoon* spp. and could remark on the possible key role domestic cats play over wild cats as reservoirs of infection (9, 12, 14). Furthermore, based on feline behavior lifestyle in environments such as colonies (49), other means of transmission, rather than traditional ones involving vectors and wild felids, could be hypothesized to contribute to the spread of *Cytauxzoon* spp. infection in domestic cats. We suspected that the close co-existence between cats in colonies is characterized by friendly and agonistic behaviors and could create a favorable environment for horizontal transmission of the parasite by blood. In fact, infection and parasitemia, without clinical signs, were experimentally obtained by inoculating blood infected with *C. manul* from Pallas' cats (*Otocolobus manul*) to cats (50). Furthermore, since blood transfusions could represent a route of transmission, as a precaution, only cats negative for PCR were recommended as donors in endemic areas (23, 51, 52). Vertical transmission is another possible way for *Cytauxoon* spp. to spread in a colony setting where, despite spay/neuter control programs, mating continues to occur. However, vertical transmission has not been fully confirmed even if young cats infected with *Cytauxzoon* spp. (23) and *C. felis* (53) were reported. Future studies are needed to better understand the main ways of transmission of *Cytauxzoon* spp. in cats, especially when they live in a socially complex society as a colony.

A limitation of the present work is that we have not performed a more in-depth molecular study than the routine PCR analysis and sequencing used to make the diagnosis. A molecular analysis of the 18S rRNA gene was completely evaluated only in the most recent samples, and it was not possible to evaluate other genes such as CytB and COI, although it would be interesting to know if the *C. europeus, C. otrantorum*, and *C. banethi* subspecies (27) were present in Italy in domestic cats. Different genotypes could vary in virulence and geographical prevalence and could cause diseases with different pathogenicity and clinical course and different responses to treatment (6, 12, 24, 54). Future studies are necessary to deepen our understanding of the genotypes present in Italy, their biological behavior, and their role in the evolution of the disease. In addition, results of biochemical and serum electrophoresis were obtained a smaller number of cats than CBC. Even if our observations appear plausible and not anecdotal, data from more subjects must be considered to find mild differences between infected and not infected cats.

## Conclusions

This study revealed that *Cytauxzoon* spp. commonly cause subclinical infections in domestic cats in Italy. In fact, no difference in clinical signs or laboratory findings was found in infected cats vs. non infected cats. Interestingly, although most of the cats were not anemic, the subclinical infected cats showed a significantly higher percentage of anemia compared to those not infected.

The infection was more likely to occur in stray cats living partially/completely outdoors, taken or recently adopted, from colonies, with irregular or absent parasite preventive treatments, without fleas and without clinical signs at the time of the medical evaluation.

## Data availability statement

The original contributions presented in the study are included in the article, further inquiries can be directed to the corresponding author. The accession number(s) can be found below: https://www.ncbi.nlm.nih.gov/genbank/, OM004051; https://www.ncbi.nlm.nih.gov/genbank/, OM004052; https://www.ncbi.nlm.nih.gov/genbank/, OM004053; https://www.ncbi.nlm.nih.gov/genbank/, OM004054; https://www.ncbi.nlm.nih.gov/genbank/, OM004055; https://www.ncbi.nlm.nih.gov/genbank/, OM004056; https://www.ncbi.nlm.nih.gov/genbank/, OM004057.

## Ethics statement

Ethical review and approval and written informed consent from the owners of the animals was not required for the study on animals in accordance with the local legislation and institutional requirements.

## Author contributions

EC: study design, sample collection, data analysis and interpretation, statistical analysis, and writing manuscript. LS-G: study design, data analysis and interpretation, and revised manuscript. SD: molecular biology and phylogenetic analysis and interpretation. LV: statistical analysis. EL: molecular biology sample processing. TF: data analysis and interpretation and revised manuscript. All authors contributed to the article and approved the submitted version.

## Dedication

This paper is dedicated to the memory of Dr. Marco Caldin (1961–2022), a mentor and a great teacher to all of us.

## Conflict of interest

The authors declare that the research was conducted in the absence of any commercial or financial relationships that could be construed as a potential conflict of interest.

## Publisher's note

All claims expressed in this article are solely those of the authors and do not necessarily represent those of their affiliated organizations, or those of the publisher, the editors and the reviewers. Any product that may be evaluated in this article, or claim that may be made by its manufacturer, is not guaranteed or endorsed by the publisher.
